# The Effect of Aquatic Intervention on the Gross Motor Function and Aquatic Skills in Children with Cerebral Palsy

**DOI:** 10.2478/v10078-012-0033-5

**Published:** 2012-05-30

**Authors:** Lidija Dimitrijević, Marko Aleksandrović, Dejan Madić, Tomislav Okičić, Dragan Radovanović, Daniel Daly

**Affiliations:** 1 Faculty of Medicine, Deptartment of Physical Medicine and Rehabilitation, University of Niš, Niš, Serbia.; 2 Faculty of Sport and Physical Education, Deptartment of Aquatic Sports, University of Niš, Niš, Serbia.; 3 Faculty of Sport and Physical Education, Deptartment of Biomedical Sciences, University of Niš, Niš, Serbia.; 4 Faculty of Kinesiology and Rehabilitation Science, Department of Kinesiology, Catholic University of Leuven, Leuven, Belgium.

**Keywords:** aquatherapy, experiment, follow up, pediatrics

## Abstract

The objective of this study was to investigate the effect of an aquatic intervention on the gross motor function and aquatic skills of children with cerebral palsy (CP). Twenty-nine children with CP, aged 5 to 14, were recruited. Fourteen children completed an aquatic intervention (EG), and 13 children served as controls (CG). Two participants dropped out due to events (illness) unrelated to the intervention. The aquatic intervention lasted 6 weeks (2 sessions per week at 55 minutes per session) with a follow-up period of 3 weeks. The outcome measures were the Gross Motor Function Measure (GMFM) for motor function and the Water Orientation Test Alyn 2 (WOTA 2) for aquatic skills assessment. A significant improvement was observed in the secondary assessment of GMFM and WOTA 2. In contrast to the aquatic skills improvement, the GMFM change was not maintained at follow-up. Our results indicate that children with CP can improve gross motor function on dry land and aquatic skills with a 6-week water intervention. The intervention period was too short for sustainable improvement in dry-land motor skills after intervention (follow-up), but time was sufficient to achieve sustainable improvements in aquatic skills.

## Introduction

Cerebral palsy (CP) is the most common physical disability in childhood (Rosenbaum, 2003; Dimitrijević et al., 2007). CP is characterised by aberrant control of movements and positions and is a consequence of early (pre-, peri- or postnatal) brain damage or dysfunction. Motor disorders in CP are the result of neurological deficit and include neuromuscular and musculoskeletal disorders: abnormal muscle tone, abnormal muscle contraction (spasticity, dyskinesia, dystonia and athetosis), bone abnormalities (foot deformities, subluxation and dislocation of the hip, long bone torsion strain), balance disorders and loss of selective motor control (Flett, 2003; Kriger, 2006).

Physical therapy (PT) plays a central role in managing the condition by focusing on function, movement, and optimal use of the child’s potential. PT uses physical approaches to promote, maintain and restore physical, psychological and social well-being (Anttila et al., 2008). Numerous therapeutic interventions have been used to minimise the development of secondary problems (normalising tone, increasing active range of motion), to improve muscle strength and mobility, to obtain functional motor skills and to encourage functional independence at home, at school and in the community (Declerck, 2010).

Aquatic intervention is one of the most popular supplementary treatments for children with neuro-motor impairments, particularly children with CP (Getz, 2006). The intervention may provide safe and beneficial alternative low-impact exercise for children with disabilities (Fragala-Pinkham et al., 2008), but there is a lack of evidence-based studies documenting the effects (Declerck, 2010).

Water is an equalising medium; its gravity-minimising nature reduces compressive joint forces, providing a better exercise environment for patients with arthritis, back pain, osteoporosis, or other medical conditions that may restrict physical training on land (Takeshima et al., 2002). Adapted aquatic exercises have been particularly recommended as a part of physical activity programs for children with CP. The buoyant nature of water provides persons with CP patients the opportunity to feel their body free from the constraints they experience on land (Getz et al., 2007; Kelly and Darrah, 2005). Water-based activity aids in the relief of pain and muscle spasms, maintenance or increases of range of motion, strengthening of weak muscles, reeducation of paralysed muscles, improvement of circulation, lung function, and speech as well as aiding in the maintenance and improvement of balance, coordination and posture (Cole and Becker, 2004).

These characteristics may allow children with CP to exercise in water with more freedom than on land. Weight relief and ease of movement allows safe movement exploration, strengthening and functional activity training with a reduced level of joint loading and impact, providing a gentler environment for children who experience persistent abnormal loading (Kelly and Darrah, 2005; Cole and Becker, 2004). In addition, aquatic physical activities are important for the teaching-learning process and might promote greater independence, better manual ability and, as a consequence, increase social participation by individuals with CP (Aidar et al., 2007).

The purpose of this study was to investigate the effect of an aquatic intervention on the gross motor function and aquatic skills of children with CP.

## Matherial and Methods

### Participants

Children with CP aged 5 to 14 years were recruited for the study through MD practitioners from the University Clinical Centre Physical Medicine and Rehabilitation Clinic Paediatric Department and the City Society of CP (Niš, Serbia). All children met the following inclusion criteria: (1) age between 5 and 14 years, (2) ability to understand instructions, (3) no medical contra-indications, (4) no botulinum toxin treatment or surgery in the preceding three months and (5) written parental approval.

An invitation letter was distributed through paediatric physiotherapists across the cities of Niš, Prokuplje, Merošina and Aleksinac, the University Clinical Centre Physical Medicine and Rehabilitation Clinic Paediatric Department and the City Society of (Niš, Serbia).

**Figure 1:**
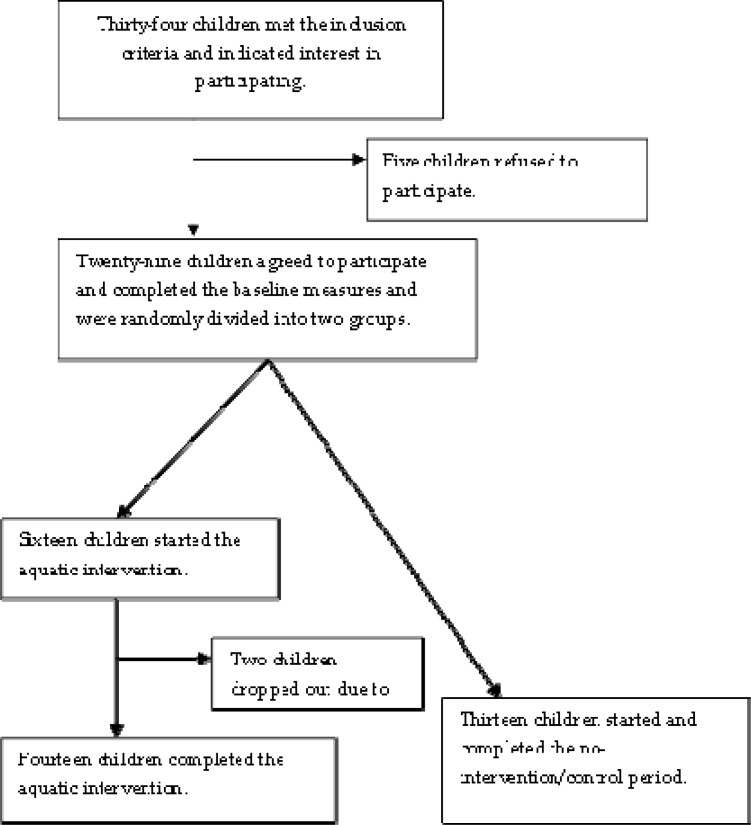
Design and flow of participants throughout the trial

A telephone interview was conducted with 34 children who met the inclusion criteria and showed an interest in participation. Five children refused to participate because of lack of time, transportation difficulties, or a time-consuming school program. Twenty-nine children agreed to participate, completed the baseline measures and were randomly divided in two groups. Fourteen children completed the aquatic intervention (EG), and 13 children completed all measurements but only participated as the no-intervention/control group (CG). After starting the intervention, 2 children had to stop due to illness (Figure 1).

All parents and children had the procedures explained to them and were asked to provide informed consent prior to data collection. The study was conducted in accordance with the Helsinki Declaration of 1975, as revised in 2000 (World Medical Association Declaration of Helsinki, 2000).

### Measures

Descriptive measures and characteristics, such as age, gender, body weight, length, type of CP and the expanded and revised Gross Motor Function Classification Scale level (GMFCS), were recorded for all study participants. GMFCS scores were used to determine the child’s present abilities and limitations in gross motor function. This classification system has been shown to be valid (Palisano et al., 2008, Bodkin et al., 2003).

The primary outcome measures were the Gross Motor Function Measure 88 (GMFM-88) for motor function and the Water Orientation Test Alyn 2 (WOTA 2) for aquatic skills assessment.

### Gross Motor Function Measure–88 (GMFM-88)

The GMFM-88 is a standardised 88-item observational instrument developed to measure changes in gross motor function over time. The test was conducted as described in the GMFM-88 manual and was performed without any aids. A percentage score was calculated for the total score as % of the five dimensions (PTS). This measure has been found to be reliable and valid (Russell et al., 2002).

### Water Orientation Test Alyn 2 (WOTA 2)

The aim of this evaluation is to assess the swimmer’s level of adjustment and function in water. The evaluation is based on the Halliwick concept, with a 10-point program subdivided into several skills. Both the swimmer and instructor were in the water at the time of testing. In addition to verbal instruction, the instructor demonstrated the task to be performed. Each item was attempted up to three times. A 4-point ordinal scale was developed for each skill based on the level of performance and functional independence. When there was uncertainty as to which score to assign, the lower of the two possible scores was chosen. The scale has been demonstrated to be reliable and valid (Tirosh et al., 2008). The following measures are calculated: mental adaptation (WMA), skills balance control movement (WSBM) and total score (WTOT).

### Procedures

The participants were enrolled in an intensive swimming program for 6 weeks (55-minutes session, 2 sessions/week) in the swimming pool of the Sports Centre “Čair” in Niš, Serbia (water temperature 27.7°C, water depth 70 cm for a 10 m x 10 m area and 180 cm for a 20 m x 10 m area).

The main objective of the swimming program was to improve safety and functional independence in the water. Each participant was taught by one instructor. The main investigator performed the aquatic therapy with the assistance of 3 additional instructors.

The aquatic therapy consisted of 10 minutes of light warm-up in the water (forward and backward walking, jumping, and other such exercises), 40 minutes of exercise swimming techniques (prone and back gliding from the wall; prone and back floating; blowing bubbles; breast-stroke, backstroke or freestyle techniques; diving to the pool bottom) and 5 minutes of play (ball games, chasing games, etc.)

The therapy was focused and performed individually. To minimise the drop-out rate, the intervention was customised to maximise enjoyment by each individual child. Depending on the spontaneous swimming technique demonstrated by each child and related functional ability, the respective child performed more breaststroke than crawl stroke or vice versa. In addition, some interventions focused more on arm movements than on leg movements and vice versa. A diary was kept to record each swimming lesson for each child separately. Thus, the goals and progression of each child could be followed intensively and individually, and every instructor was able to easily continue onto the next lesson with each child.

### Analysis

Statistical processing of all parameters was performed by calculating the mean values and standard deviation, while statistical significance (p < 0.05) was determined by Student’s t-tests. Statistical analysis was performed with SAS version 9.1.3. All measurement were repeated at the beginning and end of intervention and after 3 weeks of follow-up after cessation of intervention.

## Results

The descriptive participant data (whole sample, EG and CG) are presented in table 1. The EG consisted of 14 children (10 boys and 4 girls), and the CG was comprised of 13 children (7 boys and 6 girls).

There was no statistically significant differences between EG and CG in age (EG: 9.21 years ± 2.45, CG: 9.92 years ± 2.32), weight (EG: 29.20kg ±9:48, CG: 28.60kg ±10.04) and height (EG: 134.50cm ±13:26, CG: 136.77cm ±13.08). The EG consisted of 2 children with spastic hemiplegia, 3 with spastic diplegia, 6 with spastic quadriplegia and 3 with spastic hemiparesis, whereas the CG was made up of 2 children with spastic hemiplegia, 3 with spastic diplegia, 7 with spastic quadriplegia and one with spastic hemiparesis. The EG had 6 participants with a GMFCS score of I, 3 with GMFCS II, 2 with GMFCS III, 1 with GMFCS IV and 2 with GMFCS V, whereas the CG had 4 subjects with a GMFCS score of I, 3 with GMFCS II, 2 with GMFCS III, 1 with GMFCS IV and 3 with GMFCS V.

There was no difference in GMFM score (post-treatment score, PTS) between the groups at the start of the intervention (Table 2). By the second time point, after 6 weeks of aquatic treatment in the EG and after the same period of time of sedentary activities in the CG, there was a significantly different advantage for the EG. In the second test, which took place after a 6-week aquatic intervention, there was a statistically significant improvement in GMFM, as measured by the PTS, when compared with the initial score. In addition, there was a significant improvement in all variables related to water orientation: WMA, WSBM and WTOT (p < 0.01). In the three-week follow-up period between the second and third tests, there were no statistically significant differences in PTS or WOTA scores (Table 3).

For the CG, there were no statistically significant changes in GMFM (the CG was not included in the aquatic exercise program) (Table 2).

## Discussion

Aquatic physical activity has strong potential to benefit children with CP (Gerter and Currie, 2011). Despite the fact that swimming is one of the most frequently reported physical activities in children and adolescent with CP (Gerter and Currie, 2011), there is no consensus on optimal concepts of aquatic physical activity regarding duration of intervention period, duration of a single treatment, frequency per week of treatment, individual/group work, water temperature, swimming pool size and depth.

In our study, where the purpose was to investigate the effect of an aquatic intervention on the gross motor function and aquatic skills of children with CP, aquatic physical activity was performed for 6 weeks, twice a week, with a one to one teacher–participant ratio, in 55 min sessions, 27.7°C water temperature, and water depth between 70 cm and 180cm.

The PTS GMFM value, which represents the motor function of children with CP after 6 weeks of aquatic treatment, presented a statistically significant improvement in the EG compared with the initial measured value (p < 0.05). This result is not consistent with those of Getz (7) perhaps because a group treatment was dominant. However, this result agrees with those of Mackinnon (1997) and Troup et al. (2005), as these interventions were individually focused. Individualisation is important when working with persons with a disability and especially children with CP (Bax et al., 2005). Some studies describe the effects of individualised aquatic treatments that might be beneficial in ensuring proper technique and intensity (Kelly and Darrah, 2005).

In contrast, with the CG, there were no considerable changes in PTS value at any of the three time points; this is not unexpected with the sedentary lifestyle associated with many of the children.

In the follow-up study (no aquatic treatment), the EG PTS directly decreased, but the difference was not statistically significant. This result indicates that permanent involvement in physical therapeutic activity is necessary for children with CP to ensure that their motor functions can be maintained at a higher level. Any benefits from the aquatic intervention and any other physical activity for children with CP appears to be reduced or lost after completion of the programme, suggesting that maintenance programmes may need to be implemented for long-term benefit. Each pause in physical activity can lead to decreased motor function as a consequence (Verschuren et al., 2007; Bania et al., 2011).

An important aspect of this study is the fact that it was done using feasible and practical outcome measure in water such WOTA. This has been missing in the past (Gerter and Currie, 2011).

The initial water orientation test with the EG subjects indicated modest aquatic experience, i.e., swimming experience. However, a positive progression was evident between the first and second treatments, as measured by water orientation indicators (WMA, WSBM and WTOT) after a 6-week aquatic intervention programme (P<0.01). Individual work and adaptation for each participant positively influences movement (Čoh et al., 2004).

These results match the results of a similar study and treatment (Declerck, 2010), as well as the results of the study of Getz (2006), where improvement was evident in an aquatic skills assessment, the Aquatic Independence Measure (AIM) test, after a longer treatment (32 sessions for 16 weeks). In addition, these results are in agreement with the findings of Mackinnon (1997) and Hutzler et al. (1998a, 1998b).

In the three-week follow-up period (no aquatic treatment), there was no progression in water orientation skills; however, the skills values remained unchanged and did not decrease. These results indicate that a good motor basis in water was established for these children, which could have positive influence on motor function, other functional abilities and the quality of life (Anttila et al., 2008; Declerck, 2010; Getz, 2006; Fragala-Pinkham et al., 2008; Kelly and Darrah, 2005; Hutzler et al., 1998a; Hutzler et al., 1998b; Ballaz et al., 2010; Dorval et al., 1996; Chrysagis et al., 2009). As water orientation is a skill, it will not be forgotten after only a short time period, but motor function will vary by the time and effort put into the underlying skills training.

## Conclusion

The present study found significant effects following a 6-week aquatic intervention on the gross motor function of children with CP. Significant improvement in water skills was also observed. The intervention period was too short for sustainable improvement in dry-land motor skills after intervention (follow-up), but there was enough time to achieve sustainable improvements in aquatic skills. Future studies with a larger sample size and longer and more intensive interventions are needed.

Aquatic activities not only have a therapeutic effect on children with CP (decreasing muscle tonus, increasing motor function, increasing walking efficiency, functional abilities…), but they also have a psycho-social effect (increasing quality of life, life habits, socialization…) (Getz et al., 2007). So, in future studies researchers should use adequate questionnaires, tests or interviews for analysing these types effects.

Our study also included the highest number of participants of any other recent study (Gerter and Currie, 2011). Nevertheless, to obtain better information larger numbers are needed in the future.

## Figures and Tables

**Table 1:** Descriptive data of study participants (whole sample, experimental group and control group)

**Baseline descriptor**	**Total**	**EG**	**CG**
**Number of participants**	27	14	13
**Sex**			
Male	17	10	7
Female	10	4	6
**Age (years)**			
Mean (SD)	9.56 (2.37)	9.21 (2.45)	9.92 (2.32)
**Weight (kg)**			
Mean (SD)	28.95 (9.57)	29.20 (9.48)	28.60 (10.04)
**Height (m)**			
Mean (SD)	135.59 (12.97)	134.50 (13.26)	136.77 (13.08)
**Sub - CP Type (number)**			
Spastic Hemiplegia	4	2	2
Spastic Diplegia	6	3	3
Spastic Quadriplegia	13	6	7
Spastic Hemiparesis	4	3	1
**GMFCS**			
I	10	6	4
II	6	3	3
III	4	2	2
IV	2	1	1
V	5	2	3

**Table 2:** GMFM % scores for baseline, 6-week and 9-week time points

	**Baseline**		**6 weeks**		**9 weeks**	

	**EG**	**CG**	**EG**	**CG**	**EG**	**CG**
**PTS**	73.53 (25.63)	65.99 (29.61)	77.92 (23.63)^*^	66.56 (29.84)	73.04 (27.44)	66.56 (29.84)

Mean (SD),

* significant difference on 95% level between baseline and 6-week time points

**Table 3:** WOTA scores for baseline, 6-week and 9-week time points (only EG)

	**Baseline**		**6 weeks**		**9 weeks**	

	**EG**	**CG**	**EG**	**CG**	**EG**	**CG**
WMA	20.71 (10.82)	NA	31.93 (9.10) ^**^	NA	31.93 (9.10)	NA
WSBM	1.79 (5.32)	NA	15.57 (16.53) ^**^	NA	15.57 (16.53)	NA
WTOT	22.50 (14.25)	NA	47.50 (22.51) ^**^	NA	47.50 (22.51)	NA

*Mean (SD), WMA – Mental adaptation, WSBM - Skills balance control movement, WTOT – Total score*,

** significant difference at 99% level between baseline and 6-week time points
